# Prop-2-ynyl 3-meth­oxy-4-(prop-2-yn­yloxy)benzoate

**DOI:** 10.1107/S2414314624001639

**Published:** 2024-02-27

**Authors:** Cresten Moodley, Alfred Muller, Yonas H. Belay

**Affiliations:** a Synthesis and Catalysis Research Centre, Department of Chemical Sciences, University of Johannesburg (APK Campus), PO Box 524, Auckland Park, Johannesburg, 2006, South Africa; University of Aberdeen, United Kingdom

**Keywords:** crystal structure, dimer, hydrogen bonding

## Abstract

The title compound, C_14_H_12_O_4_, comprises of two crystallographically independent mol­ecules in the asymmetric unit, linked *via* C—H⋯O inter­actions to form dimeric entities. The allylic groups are twisted out of the phenyl planes with dihedral angles varying between 7.92 (13) and 25.42 (8)°. In the crystal, the packing follows a zigzag pattern along the *c*-axis direction. The absolute configuration of the sample could not be determined reliably.

## Abstract 2

The title compound, C_14_H_12_O_4_, was prepared *via* alkyl­ation of 4-hy­droxy-3-meth­oxy­benzoic acid with propargyl bromide in the presence of K_2_CO_3_.

## Graphical abstract

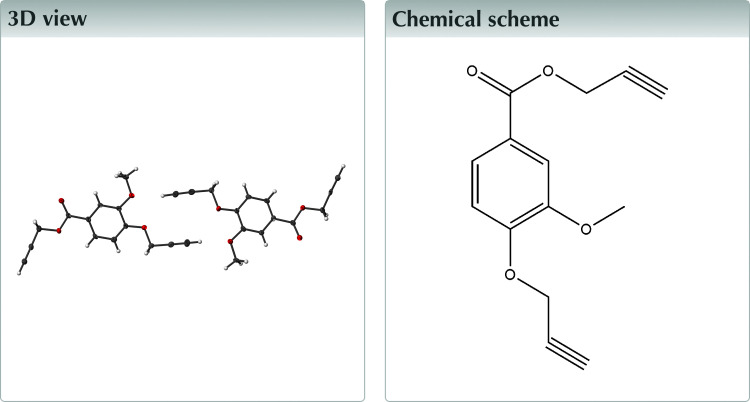

## Structure description

Vanillic acid (4-hy­droxy-3-meth­oxy­benzoic acid) is an aromatic phenolic acid widely used as a flavouring agent in the food industry. 4-Hy­droxy-3-meth­oxy­benzoic acid is naturally observed in some forms of vanilla and many other plant extracts, but may also be chemically synthesized (Calixto-Campos *et al.*, 2015 ▸). In addition to being a flavourant, this compound offers remarkable therapeutic (anti­cancer, anti­obesity, anti­diabetic, anti­bacterial, anti-inflammatory, and anti­oxidant) effects (Kaur *et al.*, 2022 ▸) and versatility for use in polymeric coatings (Silva *et al.*, 2016 ▸; El-Toni *et al.*, 2005 ▸), as an inclusion agent for encapsulants (Rajendiran & Jude Jenita, 2015 ▸; Hong *et al.*, 2008 ▸) and as a construct in metallomacrocyles (Xiong *et al.*, 2000 ▸). More recently, this compound has been reported as a promising linker precursor towards novel coordination polymers (Belay *et al.*, 2019 ▸). In this study, the title compound, C_14_H_12_O_4_, was investigated as an inter­mediate toward hydroxamic acid-type linker systems and was prepared *via* the alkyl­ation of 4-hy­droxy-3-meth­oxy­benzoic acid with propargyl bromide in the presence of K_2_CO_3_ (Buckley *et al.*, 2014 ▸; Hoogendoorn *et al.*, 2011 ▸).

**Figure 1:**
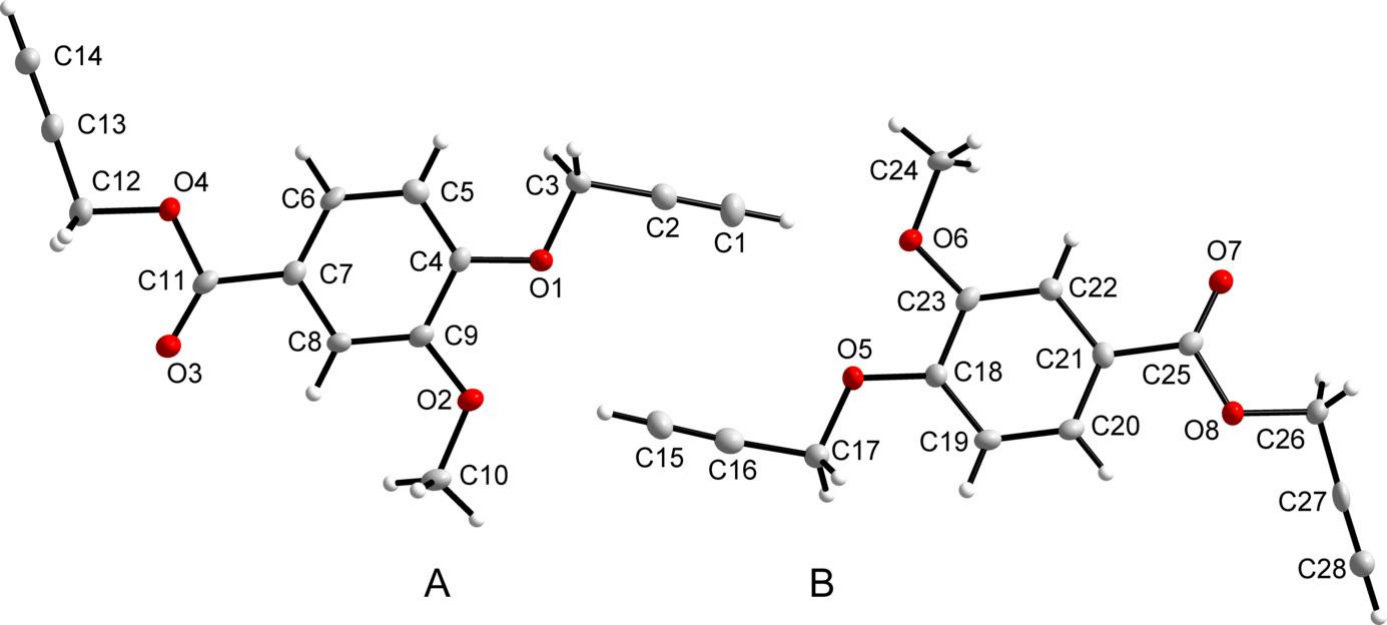
A view of the mol­ecular structure of the title compound as two independent mol­ecules (*A* and *B*) in the asymmetric unit, with the atom labelling. The displacement ellipsoids are drawn at the 50% probability level.

**Figure 2:**
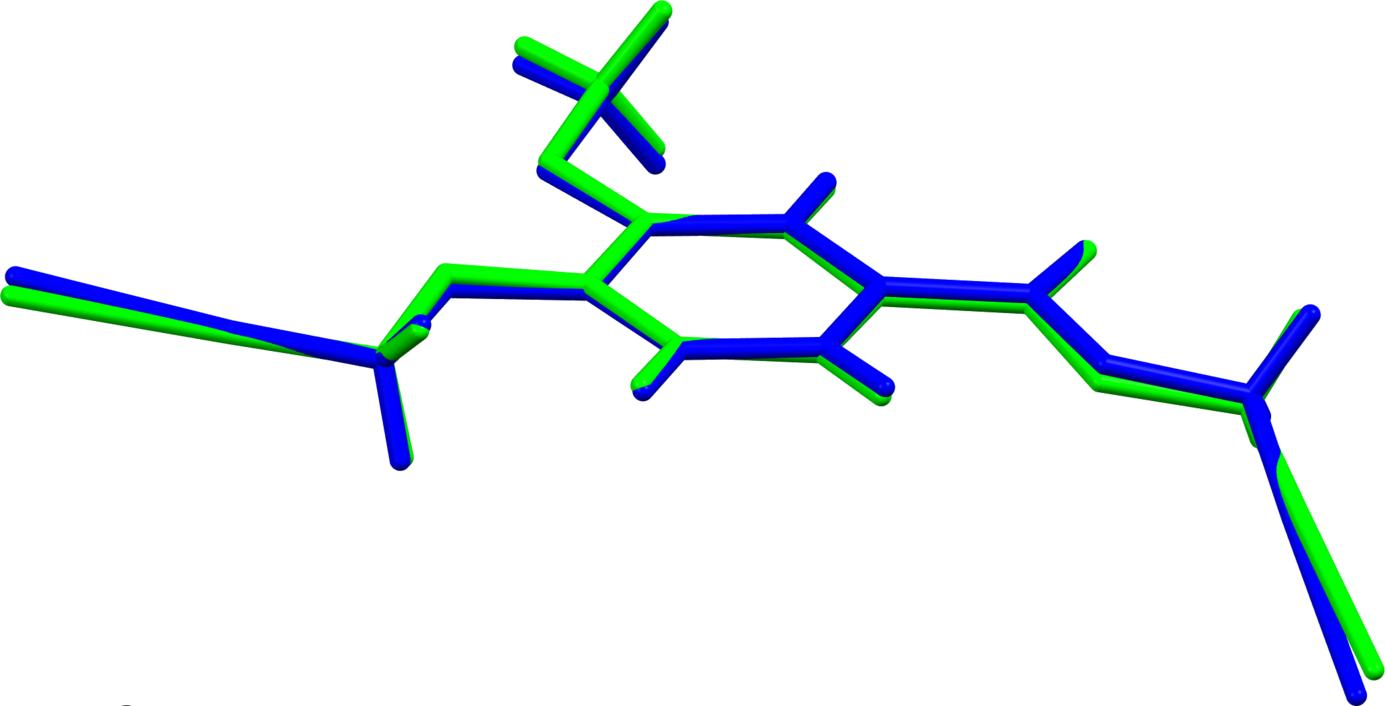
Superimposed view of the two independent mol­ecules in the asymmetric unit (r.m.s. deviation = 0.113 Å, max displacement = 0.258 Å).

The title compound crystallizes in the ortho­rhom­bic *Pca*2_1_ (*Z* = 8) space group resulting in two independent mol­ecules (*A* and *B*) in the asymmetric unit (Fig. 1 ▸) with all of the bond lengths and angles falling within the normal ranges. The differences between these two mol­ecules are observed in the allyl groups attached to the carboxyl­ate and *para*-hy­droxy positions of 4-hy­droxy-3-meth­oxy­benzoic acid, respectively, which display dihedral angles varying between 7.91 (13) and 25.42 (8)° out of plane with each of the benzoate rings for *A* and *B*. This observation is best illustrated by superimposing the two mol­ecules (Fig. 2 ▸).

In mol­ecule *A*, the dihedral angles between the benzene ring (C4–C9) and the meth­oxy group (C9/O2/C10), the ester group (O4/C12–C14) and the ether group (O1/C1–C3) are 7.17 (15), 15.80 (12) and 11.48 (15)°, respectively. In mol­ecule *B*, the corresponding angles between the benzene ring (C18–C22), the meth­oxy group (C23/O6/C24), the ester group (O8/C26–C28) and the ether group (O5/C15–C17) are 3.22 (16), 25.42 (8) and 7.92 (13)°, respectively.

**Figure 3:**
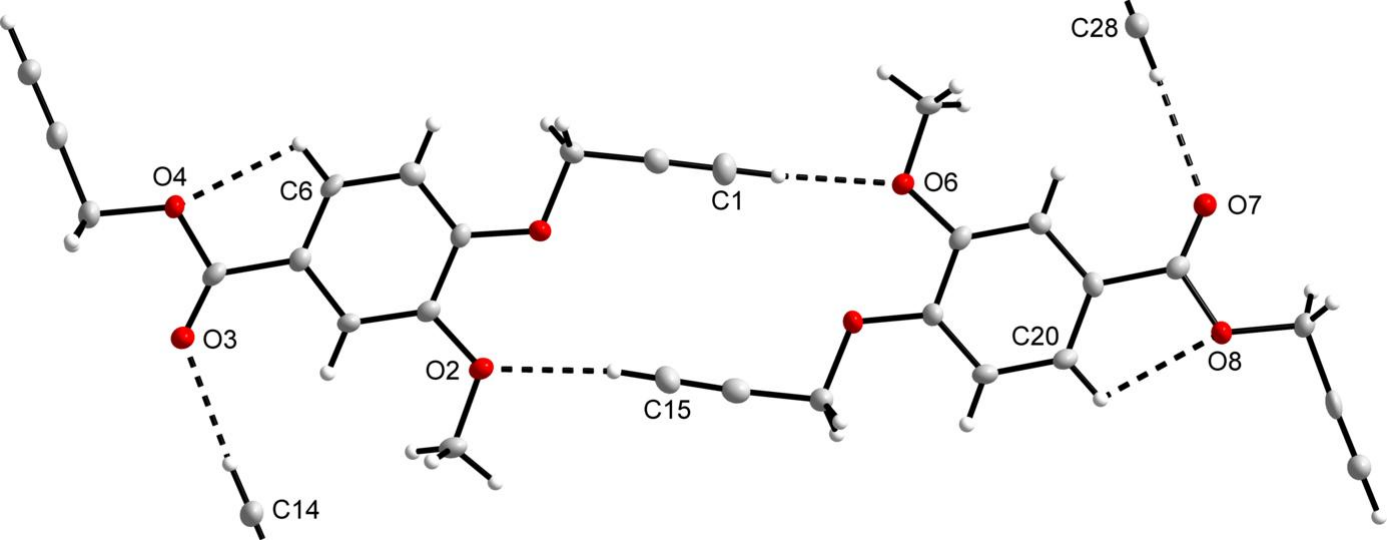
The non-classical C—H⋯O hydrogen-bonding inter­actions observed for the title compound, shown as dashed lines.

**Figure 4:**
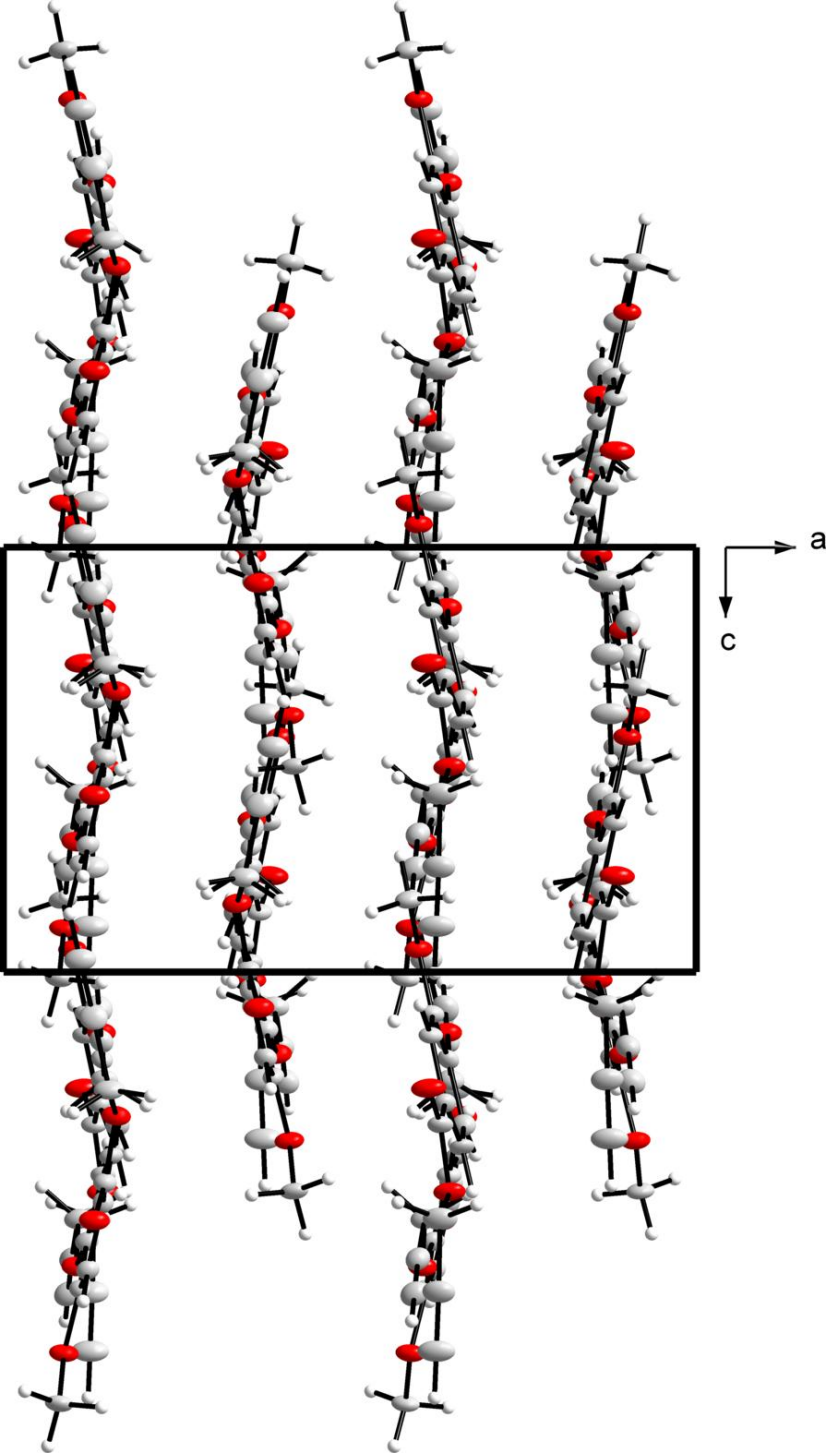
Packing diagram showing the title compound mol­ecules linked by infinite one-dimensional C—H⋯O chains along the *c*-axis direction.

Non-classical inter­molecular hydrogen bonding is observed in the extended structure of the title compound. These inter­actions (Fig. 3 ▸) are summarized in Table 1 ▸ and a packing diagram of the title compound shows the mol­ecules linked by infinite C—H⋯O chains along the *c*-axis direction (Fig. 4 ▸).

## Synthesis and crystallization

A solution of 4-hy­droxy-3-meth­oxy­benzoic acid (2.0 g, 11.90 mmol) was treated with K_2_CO_3_ (2.50 g, 17.85 mmol) in acetone. The reaction mixture was stirred under reflux for approximately 30 minutes followed by the addition of propargyl bromide (3.0 ml, 23.8 mmol of 80 wt. % in toluene). After stirring for 4 h, the reaction mixture was concentrated under vacuum. The residue was extracted using ethyl acetate, washed successively (water and brine) and dried over anhydrous sodium sulfate. The crude product was then recrystallized from the mixed solvents of di­chloro­methane and hexane to provide the title compound as colourless needles.

Analytical data: Melting point range: 65–68°C; ^1^H NMR (CDCl_3_, 400 MHZ): δ 7.69 (*d*, *J* = 8.4 Hz, 1H), 7.55 (*s*, 1H), 7.03 (*d*, *J* = 8.4 Hz, 1H), 4.88 (*d*, *J* = 2.4 Hz, 2H), 4.81 (*d*, *J* = 2.4 Hz, 2H), 3.91 (*s*, 3H), 2.52 (*t*, *J* = 2.4 Hz, 1H), 2.50 (*t*, *J* = 2.4 Hz, 1H); ^13^C NMR (CDCl_3_, 400 MHZ): δ 165.2, 150.9, 149.0, 123.4, 122.8, 112.5, 77.8, 74.8, 56.4, 55.9, 52.2.

## Refinement

Crystal data, data collection and structure refinement details are summarized in Table 2 ▸. Reflection (200) was removed as discrepant. The highest electron density of 0.22 e Å^−3^ is 0.75 Å away from C5, while the deepest electron density of −0.25 e Å^−3^ is 1.27 Å away from C25.

## Supplementary Material

Crystal structure: contains datablock(s) I. DOI: 10.1107/S2414314624001639/hb4463sup1.cif

Structure factors: contains datablock(s) I. DOI: 10.1107/S2414314624001639/hb4463Isup2.hkl

Supporting information file. DOI: 10.1107/S2414314624001639/hb4463Isup3.cml

CCDC reference: 2333706

Additional supporting information:  crystallographic information; 3D view; checkCIF report

## Figures and Tables

**Table 1:** Hydrogen-bond geometry (Å, °)

*D*—H⋯*A*	*D*—H	H⋯*A*	*D*⋯*A*	*D*—H⋯*A*
C1—H1⋯O6	0.95	2.30	3.231 (5)	165
C14—H14⋯O3^i^	0.95	2.28	3.231 (5)	177
C15—H15⋯O2	0.95	2.28	3.213 (5)	169
C28—H28⋯O7^ii^	0.95	2.41	3.291 (5)	155

Symmetry codes: (i) 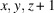; (ii) 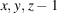.

**Table 2:** Experimental details

Crystal data	
Chemical formula	C_14_H_12_O_4_
*M*_r_	244.24
Crystal system, space group	Orthorhombic, *Pca*2_1_
Temperature (K)	100
*a*, *b*, *c* (Å)	13.7387 (12), 20.547 (2), 8.4283 (9)
*V* (Å^3^)	2379.2 (4)
*Z*	8
Radiation type	Mo *K*α
μ (mm^−1^)	0.10
Crystal size (mm)	0.40 × 0.07 × 0.03

Data collection	
Diffractometer	Bruker *APEX* DUO 4K CCD
Absorption correction	Multi-scan (*SADABS*; Krause *et al.*, 2015 ▸)
*T*_min_, *T*_max_	0.487, 0.749
No. of measured, independent and observed [*I* > 2σ(*I*)] reflections	12893, 5028, 3236
*R*_int_	0.072
(sin θ/λ)_max_ (Å^−1^)	0.670

Refinement	
*R*[*F*^2^ > 2σ(*F*^2^)], *wR*(*F*^2^), *S*	0.052, 0.111, 1.00
No. of reflections	5028
No. of parameters	326
No. of restraints	1
H-atom treatment	H-atom parameters constrained
Δρ_max_, Δρ_min_ (e Å^−3^)	0.22, −0.25
Absolute structure	Flack *x* determined using 833 quotients [(*I*^+^)−(*I*^−^)]/[(*I*^+^)+(*I*^−^)] (Parsons *et al.*, 2013 ▸)
Absolute structure parameter	−1.6 (10)

Computer programs: *APEX2* (Bruker, 2011 ▸), *SAINT* and *XPREP* (Bruker, 2008 ▸), *SIR97* (Altomare *et al.*, 1999 ▸), *SHELXL2019/2* (Sheldrick, 2015 ▸), *DIAMOND* (Brandenburg & Putz, 2005 ▸) and *WinGX* publication routines (Farrugia, 2012 ▸).

## full crystallographic data

### Prop-2-ynyl 3-methoxy-4-(prop-2-ynyloxy)benzoate Crystal data

**Table:**

C_14_H_12_O_4_	*D*_x_ = 1.364 Mg m^−^^3^
*M**_r_* = 244.24	Mo *K*α radiation, λ = 0.71073 Å
Orthorhombic, *P**c**a*2_1_	Cell parameters from 13397 reflections
*a* = 13.7387 (12) Å	θ = 0.1–28.4°
*b* = 20.547 (2) Å	µ = 0.10 mm^−^^1^
*c* = 8.4283 (9) Å	*T* = 100 K
*V* = 2379.2 (4) Å^3^	Needle, colourless
*Z* = 8	0.40 × 0.07 × 0.03 mm
*F*(000) = 1024	

### Prop-2-ynyl 3-methoxy-4-(prop-2-ynyloxy)benzoate Data collection

**Table:**

Bruker APEX DUO 4K CCD diffractometer	5028 independent reflections
Radiation source: Sealed tube	3236 reflections with *I* > 2σ(*I*)
Detector resolution: 8.4 pixels mm^-1^	*R*_int_ = 0.072
φ and ω scans	θ_max_ = 28.4°, θ_min_ = 1.0°
Absorption correction: multi-scan (SADABS; Krause *et al.*, 2015)	*h* = −18→16
*T*_min_ = 0.487, *T*_max_ = 0.749	*k* = −23→27
12893 measured reflections	*l* = −7→11

### Prop-2-ynyl 3-methoxy-4-(prop-2-ynyloxy)benzoate Refinement

**Table:**

Refinement on *F*^2^	Hydrogen site location: inferred from neighbouring sites
Least-squares matrix: full	H-atom parameters constrained
*R*[*F*^2^ > 2σ(*F*^2^)] = 0.052	*w* = 1/[σ^2^(*F*_o_^2^) + (0.0426*P*)^2^] where *P* = (*F*_o_^2^ + 2*F*_c_^2^)/3
*wR*(*F*^2^) = 0.111	(Δ/σ)_max_ = 0.003
*S* = 1.00	Δρ_max_ = 0.22 e Å^−^^3^
5028 reflections	Δρ_min_ = −0.25 e Å^−^^3^
326 parameters	Absolute structure: Flack *x* determined using 833 quotients [(*I*^+^)-(*I*^-^)]/[(*I*^+^)+(*I*^-^)] (Parsons *et al.*, 2013)
1 restraint	Absolute structure parameter: −1.6 (10)

### Prop-2-ynyl 3-methoxy-4-(prop-2-ynyloxy)benzoate Special details

**Table:**

Geometry. All esds (except the esd in the dihedral angle between two l.s. planes) are estimated using the full covariance matrix. The cell esds are taken into account individually in the estimation of esds in distances, angles and torsion angles; correlations between esds in cell parameters are only used when they are defined by crystal symmetry. An approximate (isotropic) treatment of cell esds is used for estimating esds involving l.s. planes.
Refinement. The hydrogen atoms were refined isotropically in their idealized geometrical positions while riding on their anisotropic parent atoms with *U*_iso_ = 1.2*U*_eq_ for the aromatic and methine protons, and *U*_iso_(H) = 1.5*U*_eq_(C) for the methyl protons, the latter groups were refined as a fixed rotor and adjusted to match the hydrogen atoms electron density from the Fourier difference map.

### Prop-2-ynyl 3-methoxy-4-(prop-2-ynyloxy)benzoate Fractional atomic coordinates and isotropic or equivalent isotropic displacement parameters (Å^2^)

**Table:**

	*x*	*y*	*z*	*U*_iso_*/*U*_eq_	
C1	0.8623 (3)	0.4803 (2)	0.5854 (5)	0.0277 (10)	
H1	0.865272	0.441594	0.523834	0.033*	
C2	0.8586 (3)	0.5283 (2)	0.6621 (5)	0.0216 (9)	
C3	0.8542 (3)	0.58880 (19)	0.7541 (5)	0.0218 (9)	
H3A	0.792808	0.590809	0.815537	0.026*	
H3B	0.909534	0.591193	0.829057	0.026*	
C4	0.8578 (3)	0.70289 (19)	0.7050 (4)	0.0172 (8)	
C5	0.8355 (3)	0.7179 (2)	0.8601 (5)	0.0218 (9)	
H5	0.818301	0.684249	0.932124	0.026*	
C6	0.8381 (3)	0.78228 (18)	0.9115 (4)	0.0201 (9)	
H6	0.822973	0.792274	1.018751	0.024*	
C7	0.8625 (3)	0.8317 (2)	0.8079 (4)	0.0184 (9)	
C8	0.8831 (3)	0.8172 (2)	0.6488 (4)	0.0178 (9)	
H8	0.898778	0.851112	0.576445	0.021*	
C9	0.8806 (3)	0.7534 (2)	0.5981 (4)	0.0189 (9)	
C10	0.9142 (3)	0.78343 (19)	0.3294 (5)	0.0242 (9)	
H10A	0.969498	0.810891	0.359604	0.036*	
H10B	0.855279	0.810159	0.322386	0.036*	
H10C	0.927149	0.763268	0.226153	0.036*	
C11	0.8692 (3)	0.9007 (2)	0.8589 (5)	0.0212 (9)	
C12	0.8697 (3)	0.9719 (2)	1.0807 (5)	0.0299 (11)	
H12A	0.931613	0.990641	1.041645	0.036*	
H12B	0.815584	1.000530	1.047077	0.036*	
C13	0.8716 (3)	0.9661 (2)	1.2525 (5)	0.0278 (10)	
C14	0.8750 (3)	0.9624 (2)	1.3921 (5)	0.0325 (11)	
H14	0.877700	0.959484	1.504519	0.039*	
C15	0.9067 (3)	0.5981 (2)	0.2571 (5)	0.0260 (9)	
H15	0.910724	0.635494	0.322918	0.031*	
C16	0.9016 (3)	0.5515 (2)	0.1751 (5)	0.0239 (9)	
C17	0.8920 (3)	0.49179 (19)	0.0811 (5)	0.0226 (9)	
H17A	0.829561	0.491684	0.022371	0.027*	
H17B	0.945988	0.488156	0.003873	0.027*	
C18	0.8798 (3)	0.3775 (2)	0.1339 (4)	0.0177 (9)	
C19	0.8565 (3)	0.3629 (2)	−0.0221 (4)	0.0203 (9)	
H19	0.848605	0.396833	−0.097637	0.024*	
C20	0.8446 (3)	0.29830 (19)	−0.0673 (5)	0.0197 (9)	
H20	0.828592	0.288233	−0.174173	0.024*	
C21	0.8561 (3)	0.24841 (19)	0.0419 (5)	0.0175 (8)	
C22	0.8783 (3)	0.2632 (2)	0.2010 (5)	0.0183 (9)	
H22	0.885351	0.229252	0.276632	0.022*	
C23	0.8897 (3)	0.3272 (2)	0.2463 (4)	0.0186 (8)	
C24	0.9196 (3)	0.2979 (2)	0.5156 (4)	0.0224 (9)	
H24A	0.936300	0.317677	0.617786	0.034*	
H24B	0.857032	0.275307	0.524575	0.034*	
H24C	0.970282	0.266625	0.485517	0.034*	
C25	0.8537 (3)	0.1789 (2)	−0.0043 (4)	0.0199 (9)	
C26	0.8474 (3)	0.1063 (2)	−0.2232 (5)	0.0248 (9)	
H26A	0.786129	0.081748	−0.207870	0.030*	
H26B	0.900466	0.083150	−0.167331	0.030*	
C27	0.8699 (3)	0.1123 (2)	−0.3932 (5)	0.0233 (10)	
C28	0.8893 (3)	0.1151 (2)	−0.5294 (5)	0.0271 (10)	
H28	0.904872	0.117410	−0.639094	0.032*	
O1	0.8587 (2)	0.64180 (14)	0.6418 (3)	0.0215 (7)	
O2	0.90058 (19)	0.73376 (13)	0.4462 (3)	0.0223 (6)	
O3	0.8860 (2)	0.94679 (15)	0.7732 (3)	0.0289 (7)	
O4	0.8561 (2)	0.90620 (14)	1.0171 (3)	0.0245 (7)	
O5	0.89505 (19)	0.43864 (13)	0.1930 (3)	0.0218 (6)	
O6	0.91275 (19)	0.34757 (13)	0.3969 (3)	0.0224 (6)	
O7	0.8687 (2)	0.13363 (14)	0.0841 (3)	0.0246 (7)	
O8	0.83739 (18)	0.17221 (13)	−0.1617 (3)	0.0222 (6)	

### Prop-2-ynyl 3-methoxy-4-(prop-2-ynyloxy)benzoate Atomic displacement parameters (Å^2^)

**Table:**

	*U*^11^	*U*^22^	*U*^33^	*U*^12^	*U*^13^	*U*^23^
C1	0.031 (2)	0.021 (2)	0.031 (2)	−0.0013 (19)	−0.003 (2)	−0.0048 (19)
C2	0.024 (2)	0.019 (2)	0.022 (2)	−0.0013 (18)	−0.0033 (17)	0.0021 (18)
C3	0.031 (2)	0.016 (2)	0.018 (2)	−0.0008 (17)	−0.0018 (17)	0.0027 (16)
C4	0.020 (2)	0.016 (2)	0.0157 (19)	0.0005 (16)	−0.0027 (15)	−0.0032 (16)
C5	0.025 (2)	0.020 (2)	0.020 (2)	−0.0004 (17)	0.0000 (16)	0.0021 (17)
C6	0.027 (2)	0.023 (2)	0.0110 (19)	0.0011 (17)	0.0023 (15)	−0.0049 (16)
C7	0.021 (2)	0.019 (2)	0.015 (2)	0.0025 (17)	0.0001 (15)	−0.0030 (16)
C8	0.025 (2)	0.017 (2)	0.0116 (17)	0.0020 (17)	−0.0006 (15)	0.0012 (16)
C9	0.024 (2)	0.020 (2)	0.0129 (19)	0.0031 (17)	−0.0022 (16)	−0.0005 (17)
C10	0.034 (2)	0.024 (2)	0.0141 (19)	0.0052 (19)	0.0022 (17)	0.0027 (17)
C11	0.029 (2)	0.022 (2)	0.0126 (19)	0.0030 (18)	0.0000 (16)	−0.0034 (17)
C12	0.051 (3)	0.018 (3)	0.021 (2)	−0.001 (2)	0.000 (2)	−0.004 (2)
C13	0.045 (3)	0.017 (3)	0.021 (2)	−0.0024 (19)	−0.0012 (19)	−0.0043 (18)
C14	0.059 (3)	0.019 (2)	0.019 (2)	−0.001 (2)	0.000 (2)	−0.002 (2)
C15	0.033 (2)	0.019 (2)	0.026 (2)	−0.0012 (19)	0.0044 (19)	0.0004 (19)
C16	0.026 (2)	0.023 (2)	0.023 (2)	0.0020 (18)	0.0028 (17)	0.0034 (18)
C17	0.034 (2)	0.017 (2)	0.0170 (19)	0.0020 (17)	0.0018 (18)	−0.0003 (16)
C18	0.023 (2)	0.014 (2)	0.0160 (19)	0.0024 (16)	0.0014 (15)	−0.0015 (17)
C19	0.026 (2)	0.021 (2)	0.0134 (18)	0.0003 (18)	−0.0002 (16)	0.0026 (16)
C20	0.024 (2)	0.022 (2)	0.0128 (19)	−0.0022 (17)	0.0021 (15)	0.0004 (17)
C21	0.0187 (19)	0.014 (2)	0.0202 (19)	0.0011 (17)	0.0005 (15)	−0.0016 (17)
C22	0.022 (2)	0.016 (2)	0.0163 (19)	−0.0011 (16)	0.0016 (15)	0.0007 (17)
C23	0.020 (2)	0.021 (2)	0.0144 (18)	0.0009 (16)	−0.0013 (15)	−0.0039 (17)
C24	0.034 (2)	0.022 (2)	0.0118 (18)	−0.0010 (19)	−0.0026 (16)	0.0028 (16)
C25	0.023 (2)	0.021 (2)	0.015 (2)	−0.0011 (18)	−0.0005 (16)	−0.0016 (17)
C26	0.041 (2)	0.017 (2)	0.0164 (18)	−0.0010 (19)	0.0006 (17)	−0.0035 (17)
C27	0.034 (2)	0.009 (2)	0.027 (2)	−0.0001 (18)	−0.0019 (18)	−0.0042 (17)
C28	0.043 (3)	0.018 (2)	0.021 (2)	0.0003 (19)	0.0010 (18)	−0.0010 (19)
O1	0.0349 (16)	0.0147 (16)	0.0150 (14)	0.0010 (12)	−0.0007 (12)	0.0013 (12)
O2	0.0337 (16)	0.0194 (16)	0.0138 (13)	0.0032 (12)	0.0009 (11)	0.0000 (11)
O3	0.053 (2)	0.0189 (18)	0.0153 (14)	−0.0030 (14)	0.0028 (13)	−0.0003 (13)
O4	0.0438 (17)	0.0150 (16)	0.0146 (14)	−0.0022 (13)	0.0017 (12)	−0.0031 (11)
O5	0.0365 (16)	0.0124 (15)	0.0165 (13)	0.0005 (13)	−0.0019 (12)	−0.0009 (11)
O6	0.0366 (16)	0.0172 (15)	0.0135 (12)	−0.0014 (12)	−0.0023 (11)	−0.0003 (11)
O7	0.0398 (18)	0.0174 (17)	0.0165 (14)	−0.0004 (13)	−0.0008 (12)	−0.0006 (12)
O8	0.0365 (17)	0.0136 (15)	0.0165 (13)	0.0000 (12)	−0.0022 (12)	−0.0006 (12)

### Prop-2-ynyl 3-methoxy-4-(prop-2-ynyloxy)benzoate Geometric parameters (Å, º)

**Table:**

C1—C2	1.181 (6)	C15—C16	1.182 (6)
C1—H1	0.9500	C15—H15	0.9500
C2—C3	1.466 (5)	C16—C17	1.467 (6)
C3—O1	1.444 (5)	C17—O5	1.444 (5)
C3—H3A	0.9900	C17—H17A	0.9900
C3—H3B	0.9900	C17—H17B	0.9900
C4—O1	1.364 (4)	C18—O5	1.367 (5)
C4—C5	1.378 (5)	C18—C19	1.386 (5)
C4—C9	1.410 (5)	C18—C23	1.409 (5)
C5—C6	1.392 (5)	C19—C20	1.391 (6)
C5—H5	0.9500	C19—H19	0.9500
C6—C7	1.381 (5)	C20—C21	1.387 (5)
C6—H6	0.9500	C20—H20	0.9500
C7—C8	1.403 (5)	C21—C22	1.408 (6)
C7—C11	1.484 (6)	C21—C25	1.481 (6)
C8—C9	1.379 (6)	C22—C23	1.379 (6)
C8—H8	0.9500	C22—H22	0.9500
C9—O2	1.370 (4)	C23—O6	1.373 (4)
C10—O2	1.430 (4)	C24—O6	1.433 (4)
C10—H10A	0.9800	C24—H24A	0.9800
C10—H10B	0.9800	C24—H24B	0.9800
C10—H10C	0.9800	C24—H24C	0.9800
C11—O3	1.213 (5)	C25—O7	1.209 (5)
C11—O4	1.350 (4)	C25—O8	1.353 (4)
C12—C13	1.453 (6)	C26—O8	1.456 (5)
C12—O4	1.464 (5)	C26—C27	1.471 (6)
C12—H12A	0.9900	C26—H26A	0.9900
C12—H12B	0.9900	C26—H26B	0.9900
C13—C14	1.180 (6)	C27—C28	1.180 (6)
C14—H14	0.9500	C28—H28	0.9500
			
C2—C1—H1	180.0	O5—C17—H17A	110.5
C1—C2—C3	178.8 (5)	C16—C17—H17A	110.5
O1—C3—C2	106.9 (3)	O5—C17—H17B	110.5
O1—C3—H3A	110.3	C16—C17—H17B	110.5
C2—C3—H3A	110.3	H17A—C17—H17B	108.7
O1—C3—H3B	110.3	O5—C18—C19	125.4 (4)
C2—C3—H3B	110.3	O5—C18—C23	114.5 (3)
H3A—C3—H3B	108.6	C19—C18—C23	120.1 (4)
O1—C4—C5	125.3 (4)	C18—C19—C20	119.6 (4)
O1—C4—C9	115.2 (3)	C18—C19—H19	120.2
C5—C4—C9	119.4 (4)	C20—C19—H19	120.2
C4—C5—C6	120.1 (4)	C21—C20—C19	120.7 (4)
C4—C5—H5	119.9	C21—C20—H20	119.6
C6—C5—H5	119.9	C19—C20—H20	119.7
C7—C6—C5	120.5 (3)	C20—C21—C22	119.8 (4)
C7—C6—H6	119.7	C20—C21—C25	122.4 (4)
C5—C6—H6	119.7	C22—C21—C25	117.6 (3)
C6—C7—C8	119.8 (4)	C23—C22—C21	119.6 (4)
C6—C7—C11	122.3 (3)	C23—C22—H22	120.2
C8—C7—C11	117.9 (4)	C21—C22—H22	120.2
C9—C8—C7	119.5 (4)	O6—C23—C22	125.0 (4)
C9—C8—H8	120.2	O6—C23—C18	114.8 (3)
C7—C8—H8	120.2	C22—C23—C18	120.2 (4)
O2—C9—C8	124.4 (3)	O6—C24—H24A	109.5
O2—C9—C4	115.1 (4)	O6—C24—H24B	109.5
C8—C9—C4	120.5 (4)	H24A—C24—H24B	109.5
O2—C10—H10A	109.5	O6—C24—H24C	109.5
O2—C10—H10B	109.5	H24A—C24—H24C	109.5
H10A—C10—H10B	109.5	H24B—C24—H24C	109.5
O2—C10—H10C	109.5	O7—C25—O8	123.7 (4)
H10A—C10—H10C	109.5	O7—C25—C21	125.1 (4)
H10B—C10—H10C	109.5	O8—C25—C21	111.1 (4)
O3—C11—O4	123.3 (4)	O8—C26—C27	106.8 (3)
O3—C11—C7	125.8 (4)	O8—C26—H26A	110.4
O4—C11—C7	111.0 (3)	C27—C26—H26A	110.4
C13—C12—O4	106.9 (4)	O8—C26—H26B	110.4
C13—C12—H12A	110.3	C27—C26—H26B	110.4
O4—C12—H12A	110.3	H26A—C26—H26B	108.6
C13—C12—H12B	110.3	C28—C27—C26	177.9 (5)
O4—C12—H12B	110.3	C27—C28—H28	180.0
H12A—C12—H12B	108.6	C4—O1—C3	116.0 (3)
C14—C13—C12	178.4 (5)	C9—O2—C10	117.3 (3)
C13—C14—H14	180.0	C11—O4—C12	114.9 (3)
C16—C15—H15	180.0	C18—O5—C17	116.9 (3)
C15—C16—C17	176.5 (5)	C23—O6—C24	116.3 (3)
O5—C17—C16	106.1 (3)	C25—O8—C26	115.3 (3)
			
O1—C4—C5—C6	179.3 (4)	C21—C22—C23—C18	−0.4 (5)
C9—C4—C5—C6	−1.8 (6)	O5—C18—C23—O6	0.2 (5)
C4—C5—C6—C7	0.3 (6)	C19—C18—C23—O6	−179.9 (3)
C5—C6—C7—C8	1.2 (6)	O5—C18—C23—C22	−178.6 (3)
C5—C6—C7—C11	−178.0 (4)	C19—C18—C23—C22	1.4 (5)
C6—C7—C8—C9	−1.2 (6)	C20—C21—C25—O7	175.5 (4)
C11—C7—C8—C9	178.1 (3)	C22—C21—C25—O7	0.5 (6)
C7—C8—C9—O2	−179.2 (3)	C20—C21—C25—O8	−1.2 (5)
C7—C8—C9—C4	−0.3 (6)	C22—C21—C25—O8	−176.2 (3)
O1—C4—C9—O2	−0.2 (5)	C5—C4—O1—C3	−12.5 (6)
C5—C4—C9—O2	−179.3 (3)	C9—C4—O1—C3	168.5 (3)
O1—C4—C9—C8	−179.2 (3)	C2—C3—O1—C4	−178.5 (3)
C5—C4—C9—C8	1.8 (6)	C8—C9—O2—C10	−7.4 (5)
C6—C7—C11—O3	−176.6 (4)	C4—C9—O2—C10	173.7 (3)
C8—C7—C11—O3	4.2 (6)	O3—C11—O4—C12	−3.7 (6)
C6—C7—C11—O4	4.8 (5)	C7—C11—O4—C12	175.0 (3)
C8—C7—C11—O4	−174.4 (3)	C13—C12—O4—C11	−169.1 (3)
O5—C18—C19—C20	178.8 (3)	C19—C18—O5—C17	−3.7 (5)
C23—C18—C19—C20	−1.1 (6)	C23—C18—O5—C17	176.2 (3)
C18—C19—C20—C21	−0.1 (6)	C16—C17—O5—C18	175.1 (3)
C19—C20—C21—C22	1.0 (5)	C22—C23—O6—C24	−3.7 (5)
C19—C20—C21—C25	−173.9 (4)	C18—C23—O6—C24	177.6 (3)
C20—C21—C22—C23	−0.8 (5)	O7—C25—O8—C26	−5.1 (6)
C25—C21—C22—C23	174.3 (3)	C21—C25—O8—C26	171.7 (3)
C21—C22—C23—O6	−179.0 (3)	C27—C26—O8—C25	−155.7 (3)

### Prop-2-ynyl 3-methoxy-4-(prop-2-ynyloxy)benzoate Hydrogen-bond geometry (Å, º)

**Table:**

*D*—H···*A*	*D*—H	H···*A*	*D*···*A*	*D*—H···*A*
C1—H1···O6	0.95	2.30	3.231 (5)	165
C14—H14···O3^i^	0.95	2.28	3.231 (5)	177
C15—H15···O2	0.95	2.28	3.213 (5)	169
C28—H28···O7^ii^	0.95	2.41	3.291 (5)	155

Symmetry codes: (i) *x*, *y*, *z*+1; (ii) *x*, *y*, *z*−1.
